# Flint’s Encyclopedia of Medicine and Surgery

**Published:** 1898-06

**Authors:** 


FLINT'S ENCYCLOPEDIA OF MEDICINE AND SURGERY. By various writers. Second Revised Edition. 1898. J. B. Flint & Co., New York. Pages, 1558. Price, cloth, $5; half morocco, $6.

This is another production of that modern fiend, the booksmith. It is an alphabetical compilation of the writings of various authors, many of whom have been dead for years, and whose opinions are now entirely out of date. There is not even the pretense of an editor, it being frankly stated on the title page that it is "compiled under the direction of the publishers." The medical profession is patient and long suffering, and has been paying, without complaint, exhorbitant prices for its professional books, doubtless under the belief that at least a modicum of the price goes to reward the patient toil, and frequently the brilliant genius, of the learned author. But in the present case, as before stated, there is no such subterfuge. The price proposed to be charged is simply outrageous for any book of its size, without any expense of authorship. Its mechanical execution is of the cheapest style, and in this respect comports well with its contents. As a justification for the strictures in this review, and for no other purpose, attention is called to some of the contents. Asepsis is not mentioned, but several pages are devoted to antiseptic treatment, in which, among other things equally modern(?), it is stated that: "Wherever the skin is destroyed * * * inflammation and fever ensue. This is entirely due to the growth and development in the wound of secretions of micro-organisms, which *abounding* in the *atmosphere*, are deposited everywhere." And further on the surgeon is advised to perform his operations under a 1-30 carbolic acid spray. We have too much respect for the memory of the great Lister to make his ghost walk in this manner. The definition of malaria is: "An acute, non-contagious, specific disorder, produced by a *miasm, generally air-borne.*" The book is only valuable to point a moral. J.

